# Effect of Anticoagulants in Pulmonary Thromboembolism in Post-COVID-19 Patients

**DOI:** 10.7759/cureus.39382

**Published:** 2023-05-23

**Authors:** Caner Çınar, Zeynep Ceren Balaban Genç, Selin Kesim, Feyza Çağlıyan Şen, Sait Karakurt, Tanju Yusuf Erdil, Tunç Öneş, Emel Eryuksel

**Affiliations:** 1 Department of Pulmonology, Marmara University, Istanbul, TUR; 2 Department of Nuclear Medicine, Marmara University, Istanbul, TUR

**Keywords:** post-covid-19, d-dimer, anticoagulation, pulmonary thromboembolism, covid-19

## Abstract

Background and objective

COVID-19 may predispose to both venous and arterial thromboembolism (VTE and ATE) due to excessive inflammation, immobilization, and hypoxia. The purpose of this study is to evaluate clinical and laboratory risk factors, as well as related medications such as anticoagulants, to predict the risk of thromboembolic disease and/or death in COVID-19 patients.

Methods

Over a period of 14 months (from August 2020 to September 2021), a total of 145 consecutive patients with signs and symptoms suspicious of pulmonary embolism (PE) were referred for perfusion single-photon emission computed tomography/computed tomography (Q SPECT/CT). All patients had a history of SARS‑CoV‑2 infection, diagnosed with a positive real-time polymerase chain reaction (RT-PCR) test.

Results

Among the 145 patients included in the study, the risk of PE was found to be greater in elderly patients (odds ratio [OR] [95% CI]: 1.05 [1.02‑1.07]; p<0.001) and in patients with higher maximum d-dimer levels (OR [95% CI]: 1.14 [1.01‑1.3]; p=0.04). We also analyzed the utility of the maximum d-dimer level for predicting acute PE with receiver operating characteristic (ROC) curve analysis. For d‑dimer = 0.5 mg/dL, cut-off sensitivity is 91%, specificity is 23%, and for d-dimer = 1 mg/dL, cut-off sensitivity is 79%, specificity is 43%

Conclusion

D-dimer titers were higher in the PE group in our study. Another significant finding was that, possibly due to thromboinflammation, anticoagulants did not prevent the development of PE in COVID-19 patients.

## Introduction

After the outbreak of the novel coronavirus infection originated in Wuhan city of China, in the last months of 2019, WHO subsequently named the disease COVID-19, which initiated a health crisis with a phenomenal increasing trend in terms of morbidity and mortality rates [1]. COVID-19 is reported to be associated with a high rate of macrovessel thrombotic events, including venous thromboembolism (VTE) such as deep vein thrombosis (DVT) and pulmonary embolism (PE) and arterial thromboembolism (ATE) such as stroke and myocardial infarction (MI). The incidence of pulmonary thromboembolic disease (PTE) in COVID-19 patients has been reported from 14.8% up to 30% in various retrospective studies [2-4]. There is limited and conflicting data on thromboembolic events and death rates in hospitalized COVID-19 patients after discharge. Previous research has been limited by small sample sizes, retrospective designs, and non-standard follow-up procedures. On the other hand, antithrombotic guidelines cannot provide a clear recommendation for thromboprophylaxis after a prolonged hospital stay for COVID-19 patients. The purpose of this study is to evaluate clinical and laboratory risk factors, as well as related medications such as anticoagulants, to predict the risk of thromboembolic disease and/or death in COVID-19 patients.

## Materials and methods

Study subjects and patient selection

Over a period of 14 months (from August 2020 to September 2021), a total of 145 consecutive patients with signs and symptoms suspicious of pulmonary embolism (PE) were referred for perfusion single-photon emission computed tomography/computed tomography (Q SPECT/CT). All patients had a history of SARS‑CoV‑2 infection, diagnosed with a positive real-time polymerase chain reaction (RT-PCR) test. In this study, the inclusion criteria for the study were patients who had already been diagnosed with COVID-19. After the disease period, patients who exhibited suspicious symptoms, such as increased dyspnea or decreased exercise capacity, were considered. Additionally, those patients were included if non-embolic causes had been excluded by physical examination and lung tomography and/or if they had increased D-dimer levels. The patients with a known history of PE were excluded from the study. Patients who met the inclusion criteria underwent Q-SPECT/CT.

Clinical data analysis

Patients' clinical data, including demographic characteristics, COVID-19 nasopharyngeal and throat swab results, diagnosis date, d-dimer levels (d-dimer >0.5 accepted as laboratory inclusion criteria for PE suspicion), length of hospital stay, treatment type, and duration were obtained from the clinical records of the patients.

Perfusion SPECT/CT protocol and imaging analysis

Due to the high transmission risk generated by infectious aerosols during the ventilation procedure, only perfusion SPECT images with low-dose CT scans were obtained by using a 180° dual head detector on SPECT/CT (Siemens Symbia TruePoint, Siemens Medical Solutions, USA) [5]. After slowly (within 20‑30 s) injecting the doses of 4-5 millicurie (148-185 megabecquerels) of Technetium-99m macro-aggregated albumin (TechneScan LyoMAA, Mallinckrodt Medical) containing 100.000‑200.000 particles, SPECT/CT acquisition was performed (low‑energy high-resolution collimator, 128×128 matrix, 64 projections of 10 s, 1.00 zoom factor, 140±10% keV energy window). The CT parameters were 13-25 mAs, a peak tube voltage of 130 kV, and a slice width of 5 mm. Raw data of SPECT images were processed with the "Tomo Reconstruction v.8.2.26.4" (Syngo‑Siemens AG) application and iterative reconstruction was conducted with the ordered‑subset expectation maximization (OSEM) method. The diagnosis of PE was made according to the recommendations of the European Association of Nuclear Medicine (EANM) guideline [6]. Q-SPECT/CT images were evaluated by two nuclear medicine physicians blinded to patients' clinical information to reach a final consensus.

Ethics statement

This study was performed with local IRB approval (dated December 3, 2021; No: 1318) and a waiver of individual informed consent.

Statistical analysis

All statistical analyses were performed using IBM SPSS® software version 25.0. Descriptive statistics were used to present the characteristics of the study population. Median (range) was provided for continuous, non-normally distributed variables, and frequencies (percentage) were reported for categorical variables. The distribution of numerical data was determined by Kolmogorov-Smirnov and Shapiro-Wilk tests. Student t-test and Mann-Whitney U test were used to compare the differences between the groups for these variables. Categorical variables were evaluated using the Chi-squared test. Cut-off values for the d-dimer level were evaluated using ROC. Possible risk factors for PE were evaluated by logistic regression analysis. Results with 95% CI and two-sided p<0.05 were considered statistically significant.

## Results

In total, 2480 patients came to the outpatient clinic after COVID-19 infection during the study period. Among them, 145 patients who met the inclusion criteria were taken as the study population; 49% (n = 71) of the patients were referred to Q‑SPECT/CT due to elevation of d-dimer levels (>0.5 mg/dL). Thirty-seven patients (25.5%) had at least one symptom, i.e., dyspnea or chest pain, and 37 (25.5%) had both laboratory and clinical suspicion for PE. The mean time between the positive RT-PCR tests and imaging was 56.2 ± 23.7 (range 0‑145) days. When the groups with and without perfusion defect (PD) were compared, no statistically significant difference was found between gender, the reason for referral, Siddiqi classification on admission, changes in d-dimer, anticoagulant therapy usage, time and presence of PD (p>0.05). The mean age (<0.001), maximum d-dimer level in the first 15 days (p=0.007), the d-dimer level at outpatient follow-up (p=0.022), and the time of hospital stays (p=0.017) of the patients with PD were significantly higher than those with no PD on Q‑SPECT/CT images. The demographic, laboratory, and outcome data of the patients with and without PD are presented in Table 1.

**Table 1:** Patient characteristics. PD: Perfusion defect; Q-SPECT/CT; Perfusion single-photon emission computed tomography/computed tomography; PE: Pulmonary embolism; LMWH: Low-molecular-weight heparin; RT-PCR: Reverse transcription polymerase chain reaction. ^a^Student t test; ^b^Chi-squared test; ^c^Mann-Whitney U test.

	Total	No PD on Q-SPECT/CT	PD on Q-SPECT/CT	P-value
(n, % or SD)	(n, % or SD)	(n, % or SD)		
	n = 145	n = 86 (59.3)	n = 59 (40.6)	
Age	59.1 ± 15 (25-90)	55.2 ± 13.9 (26-83)	64.8 ± 14.8 (25-90)	<0.001^a^
Sex				0,529^b^
Male	79 (54.5)	45 (52.3)	34 (57.6)	
Female	66 (44.5)	41 (47.7)	25 (42.4)	
PE suspicion				0.167^b^
Laboratory (d-dimer ≥ 0.5 mg/dL)	71 (49)	42 (48.8)	29 (49.2)	
Clinical (dispnea/chest pain)	37 (25.5)	26 (30.2)	11 (18.6)	
Laboratory + Clinical	37 (25.5)	18 (20.9)	19 (32.2)	
Siddiqi classification				0,068^b^
Stage I	35 (24.1)	23 (26.7)	12 (20.3)	
Stage IIA	18 (12.4)	11 (12.8)	7 (11.9)	
Stage IIB	82 (56.6)	50 (58.1)	32 (54.2)	
Stage III	10 (6.9)	2 (2.3)	8 (13.6)	
Hospital stays (day)	10.85 ± 12.2 (0-79)	9.1 ± 11.3 (0-79)	13.3 ± 13.1 (0-67)	0.017^c^
Maximum d-dimer level	2.7 ± 3.6 (0.0-19.3)	2.07 ± 3 (0.2-19.3)	3.6 ± 4 (0.0-18.3)	0.007^c^
D-dimer level at discharge	0.85 ± 0.9 (0.0-7.7)	0.69 ± 0.5 (0.06-3.1)	1 ± 1.3 (0.0-7.8)	0.102^c^
D-dimer level at outpatient follow-up	1.46 ± 1.6 (0.13-11.2)	1.35 ± 1.8 (0.13-11.2)	1,6 ± 1.3 (0.16-5.24)	0.022^c^
Changes in d-dimer				0.196^b^
Increased	78 (80)	49 (83)	29 (77)	
Same	2 (2)	0 (0)	2 (5)	
Decreased	17 (18)	10 (17)	7 (18)	
LMWH treatment type				0.561^b^
Absent	38 (27.1)	22 (26.2)	16 (28.6)	
Once daily	84 (60)	53 (63.1)	31 (55.4)	
Twice daily	18 (12.9)	9 (10.7)	9 (16.1)	
LMWH treatment time (day)	18 ± 15.9 (0-72)	16.6 ± 14.5 (0-72)	20 ± 17.8 (0-68)	0.204^c^
RT-PCR to imaging interval (day)	56.2 ± 23.7 (0-145)	58.8 ± 22.6 (12-145)	52.4 ± 25 (0-127)	0.704^a^

**Figure 1:**
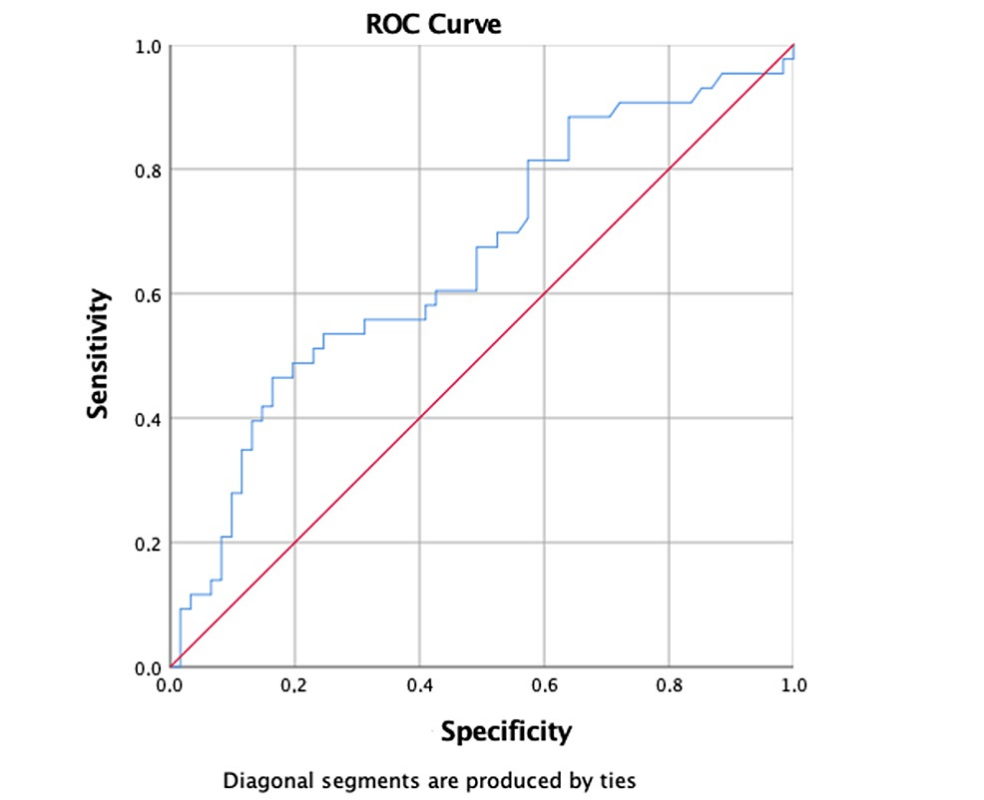
D-dimer ROC curve. ROC curve: Receiver operating characteristic curve.

The risk of PE was found to be greater in elder patients (odds ratio [OR] [95% CI]: 1.05 [1.02‑1.07]; p<0.001) and in patients with higher maximum d-dimer levels (OR [95% CI]: 1.14 [1.01‑1.3]; p=0.04). However, in multivariate logistic regression analysis, only age remained an independent risk factor for PE. Subsequently, we analyzed the utility of the maximum d-dimer level for predicting acute PE with ROC curve analysis. For d‑dimer = 0.5 mg/dL, the cut-off sensitivity is 91%, and the specificity is 23%. For d-dimer = 1 mg/dL, cut-off sensitivity is 79%, and specificity is 43%, as presented in Figure 1. 

## Discussion

The maximal d-dimer level in the first 15 days and the d-dimer level in the outpatient follow-up of PE patients were shown to be considerably high at the conclusion of this trial; nevertheless, low-molecular-weight heparin (LMWH) administration was not shown to be PE protective. PE was also more common in individuals who were older and had stayed in the hospital for a longer period of time. These findings confirm the concept that higher thrombotic events in COVID-19 are caused by thromboinflammation, with the degree of inflammation being the most relevant risk factor. Thrombotic events are frequent in COVID-19 patients admitted to the hospital and are a strong predictor of poor prognosis [7]. Lung ventilation/perfusion (V/Q) scintigraphy is a well-established imaging method specifically used for diagnosing PE. During the COVID-19 pandemic, it has been recommended to perform only perfusion scans to minimize the risk of aerosol contamination [5]. Q-SPECT/CT is reported to be an efficient imaging modality for PE diagnosis in patients during the post-COVID-19 period, particularly in those who experienced a mild-to-moderate course of COVID-19 [8]. The role of prolonged anticoagulation after hospital discharge is still a subject of debate. According to the American Society of Hematology, routine anticoagulation is not recommended for outpatients with COVID-19 [9]. The National Institutes of Health do not recommend routine anticoagulation for non-hospitalized patients. However, they recommend using these medications during hospitalization and continuing VTE prophylaxis after discharge for patients with a low risk of bleeding but a high risk of VTE [10]. One hundred two patients in this research period were administered anticoagulation at least one dose following discharge, in accordance with Ministry of Health recommendations. Prophylaxis is recommended for patients with active malignancy, immobility, a history of major surgery or embolism, obesity, and a d‑dimer level greater than twice the normal value at discharge. Patients who were hospitalized during the COVID-19 infection period received normal anticoagulant therapy during their stay and for at least 10 days after discharge. Thirty-eight patients (27.1% of the total patient population) with mild illness were not given anticoagulants on a regular basis.

However, in standard medical practice, the definition of a high-risk patient is based on the general definition of a PE at risk and does not account for any additional considerations for COVID-19 patients. Li P et al. have confirmed the low incidence of symptomatic VTE in COVID-19 patients after discharge. Their study is the first to collect enough post-discharge VTE events to determine the characteristics associated with higher risk. They discovered that individuals hospitalized with COVID-19 who had a history of VTE, a predischarge CRP level more than 10 mg/mL, or a peak D-dimer level greater than 3 g/mL during hospitalization were more likely to develop new VTE after discharge [11]. In a meta-analysis, Kwee RM et al. discovered that COVID-19 patients with PE had higher D-dimer values and that the prevalence of PE was higher in the more severe patients [12]. The other planned retrospective investigation found that the PE-positive group's d-dimer median value was considerably greater than the PE-negative group's [13]. In a retrospective study, Mouhat B et al. looked at patients who were hospitalized owing to COVID-19 infection and had confirmed diagnoses of PE on CT pulmonary angiography (CTPA) and discovered that elevated D-dimer levels and lack of anticoagulant medication could predict PE development risk [14]. Our findings are comparable to these researches in some respects but not others. In the outpatient follow-up, patients with perfusion defects on Q-SPECT/CT images had substantially higher d‑dimer and maximal d-dimer levels in the first 15 days than those without perfusion defects. Earlier researches have demonstrated that the d‑dimer level during hospitalization is a risk factor for post-discharge PE, and our findings are consistent with other studies in this regard. On the other hand, routine monitoring of d‑dimer levels during discharge is not advised. However, according to the findings of our investigation, d‑dimer levels rise dramatically in individuals with probable PE. A rise in d‑dimer levels, particularly in the post-COVID period, maybe a warning sign of PE and may necessitate further assessment. This finding has to be validated in larger-scale investigations with more patients. During the early stages of the pandemic, some meta-analyses in the literature revealed that VTE was very common in patients with COVID-19, accounting for up to 33% of patients in the ICU even when anticoagulation was used [15,16].

We also found significantly higher levels of the d-dimer level at outpatient follow-up (p=0.022) and maximum d-dimer level in the first 15 days (p=0.007) of the patients with perfusion defect in the Q-SPECT/CT images group than those with no perfusion defect. Some coagulation disorder markers (e.g., D-dimer) appear to be associated with the severity of COVID-19 disease. D-dimer values ​​may be found to be increased in people with intensive care indications and experiencing severe forms of the disease, such as disseminated intravascular coagulation (DIC). In our study, although there was no difference between the PD group and the non-PD group in terms of the Siddiqi classification (p=0.068), which is the scale showing the severity of the disease [17], the advanced age (p<0.001) and the longer hospitalization period (p=0.017) of the PD group may indirectly suggest that d-dimer values ​​are higher in the population with severe disease in our study. The severe disease could be the reason for the development of pulmonary thromboembolism in this group. There was no difference between the two groups in the Siddiqi performance scores; this could have arisen due to the retrospective nature of our study and the fact that Q-SPECT imaging of the patients is usually performed after discharge from the hospital and patients who died from COVID-19 during the hospitalization period were not included in our study group.

The fact that thrombosis is a major cause of clinical deterioration and mortality has sparked a worldwide debate on whether increasing the anticoagulant dosage or extending the duration of treatment improves patient outcomes. According to Giannis D et al., anticoagulant drugs used after discharge following COVID-19 infection reduce the risk of major thromboembolism or all-cause mortality by 46% [18]. Compared to our study, the endpoint in that study was all arterial and venous thrombosis events and mortality, and it demonstrated that anticoagulant therapy could reduce arteriovenous thromboembolic events and all-cause mortality. The reason for the ineffectiveness of prophylaxis, as observed in our study, may be that the embolisms occurring in patients are not major embolisms that cause death. In addition, the small number of samples in our study and its retrospective design may be other reasons.

In our study, there was no difference between the PD and non-PD groups in terms of the Siddiqi classification (p=0.068), a scale that shows the severity of the disease. The PD group's advanced age (p<0.001) and longer hospitalization period (p=0.017) may indirectly suggest that d-dimer values are higher in the population with severe disease in our study and that more severe disease could be the cause of the development of pulmonary thromboembolism. The Siddiqi performance scores did not differ between the two groups; this could be due to the retrospective nature of our study, as well as the fact that Q-SPECT/CT imaging of patients is usually done after discharge from the hospital, and patients who died from COVID-19 during their hospitalization were not included in our study group.

The primary cause of death in COVID-19 appears to be acute lung injury (ALI), characterized by severe inflammation, extensive thrombosis, and severe endothelial damage of perialveolar capillaries in autopsy studies [19, 20]. The reason why anticoagulant drugs cannot prevent the development of thrombosis in the course of COVID-19 may be the development of thrombosis due to thromboinflammation that develops during the course of infection. It seems that more prospective or randomized controlled studies are needed in order to make definite recommendations about anticoagulation.

Our research does have certain limitations. Because it was a single-center, retrospective study of patients who had Q-SPECT/CT, usually after hospital discharge, data from patients who had more severe diseases and died in the hospital could not be included in the analysis. Similarly, the patients' concomitant diseases and related thrombotic factors (e.g., hematology, rheumatology, immobility, past VTE history, etc.) were neglected.

## Conclusions

In conclusion, we found that d-dimer titers were higher in the PE group in our study. The mean age and hospitalization duration of the group developing pulmonary thromboembolism were significantly higher than those of the other group, suggesting that pulmonary thromboembolism occurs in more severe COVID-19 patients. Another significant finding of our study was that anticoagulants did not prevent the development of PE in COVID-19 patients. These findings may be critical in individuals with increasing D-dimer levels after discharge that cannot be explained by any other reason. More detailed multicenter cohort studies are needed for more precise results. 
